# SymPET, a waveform digitizing “System on Chip” for ultra-high resolution TOF PET: design concept and preliminary studies

**DOI:** 10.1109/nss/mic44845.2022.10398916

**Published:** 2022-11

**Authors:** K. Flood, C. Chock, L. Blackberg, Y. Feng, S. Hashemi, L. Macchiarulo, M. Mishra, D. Thelen, I. Mostafanezhad, H. Sabet

**Affiliations:** 1 Nalu Scientific LLC, Honolulu, HI, USA.; 2 Department of Radiology, Massachusetts General Hospital & Harvard Medical School, Boston, MA, USA.

**Keywords:** System-on-chip, waveform digitization, picosecond timing, TOF PET, SiPM array readout

## Abstract

SymPET is a low-power, high channel density, waveform-digitizing readout microchip under development at Nalu Scientific for SiPM-based TOF PET applications. Our “System on Chip” waveform digitizing architecture includes features such as fully random accessible analog storage, input triggering, and on-chip biasing, control and waveform feature extraction capabilities, enabling many effective mechanisms to optimize features such as e.g., throughput, speed, and buffer length while simultaneously allowing excellent control of systematic effects which typically significantly affect the timing precision of time-over-threshold based readouts. Preliminary results show that SymPET can provide less than 10 ps timing jitter at a reasonable power budget which is substantially better compared to existing solutions. This will allow for high-channel density and/or limited angle applications such as ultra high resolution brain TOF PET designs that require proper management of heat dissipation, while necessitating high spatial and timing resolution.

Advances in the design and fabrication of Application Specific Integrated Circuits (ASICs) now make it possible to develop competitively priced digitization electronics with picosecond-level timing precision [1] using low power, weight, and size ASICs that support complex on-chip data processing tasks such as waveform digitization and feature extraction. Nalu Scientific has previously designed and developed several such “System on Chip” waveform digitizing readout chips which have been successfully, or are planned to be, deployed in large high energy and nuclear physics detectors. TOF PET image quality is principally driven by system-level timing resolution, and pushing the best current CTRs significantly lower to approach ~10 ps will require matching (a) an improved very fast scintillating material(s) with good light yield for 511keV gammas, small photon transfer time spread and capable of supporting innovative geometries for arrays, with (b) high-efficiency high-channel-density low-noise SiPM photosensors and readout electronics with single photon sensitivity and precise digital time-stamping and spatial reconstruction for each signal photon. Further exploration of super-fast novel inorganic and organic scintillating materials (some of which can have physical response times approaching ~1 ps) as well as other very fast emission processes such as cross-luminescence, Cerenkov radiation in scintillating and non-scintillating materials, hot intra-band luminescence, and the prospective use of nanomaterials with tunable fundamental physical properties, will play an important role in pushing TOF PET imaging towards the goal of 10 ps timing resolution. A low-cost, commercial, modular readout solution with the physical characteristics, technical performance and features of SymPET will be required to optimally leverage this continuing evolution of ultra-fast materials and sensor technologies and fully realize the potential of TOF PET imaging.

Several techniques can be used to measure the arrival time of very fast electrical pulses, including (a) the time at which the pulse crosses a single fixed threshold or, to increase timing resolution, multiple thresholds; (b) the time at which the pulse initially reaches a constant fraction of its peak amplitude; or (c) fast analog waveform sampling into arrays of storage capacitors and subsequent digitization at rates on the order of ~10 GSa/s. [2] Each of these methods typically requires analog bandwidths at the level of ~1–2 GHz. Traditional timing methods using hardware-based single or dual thresholds to estimate the time of arrival of photons incident on a detector suffer from multiple sources of systematic timing uncertainties: a single threshold crossing causes timing precision degradation primarily from the “walk” effect since signal rise-time is not well-constrained. To mitigate this, a second threshold on the falling edge of the detector response can be added, which also returns information on the full width at half max (FWHM) and thus the total charge of a decaying signal, which can be used as an energy estimator. Full waveform digitization can be viewed as effectively implementing an arbitrary number of thresholds limited only by the signal bandwidth, effectively averaging over any noise and, through the use of on-chip waveform feature extraction, allowing real-time corrections for many of the effects contributing to timing imprecision such as e.g. timewalk, baseline wander, and waveform shape variations while also providing high-precision energy estimation. As shown in [5] it is possible to use information from a fast sampled signal to perform timing estimation at the subsample time - with a system operating at 5Gsps (½ of the sampling speed of the most advanced Nalu digitizers) it is theoretically possible to reach the limit of 1 ps. With the existing electronics developed by Nalu Scientific, we were able to demonstrate pulse-to-pulse timing around 10ps, and it is possible that better calibration method and correction will render the contribution of the frontend readout electronics to the timing estimation negligible compared to the other sources of jitter.

Fig. 1 shows the main functional blocks of Nalu’s AARDVARC chip. The signals from each channel are continuously sampled and stored in a large analog memory (labeled storage array in the figure). The readout is triggered by logic operating on the basis of the self triggers, or optionally by external logic, and is managed internally through a randomly accessible storage digitizer embedded in the array with the digital samples being transferred to a DATA RAM. The data can be immediately processed through DSP functions (basic pedestal subtraction with pedestals contained in a Pedestal RAM (The Pedestal RAM is external for the prototype but will be incorporated in future versions of the Aardvarc), and can be streamed out via a Direct memory access block (DMA) directly or more conveniently after a feature extraction algorithm under the control of a general CPU, that is also the master controller. Large flexibility is possible by flushing customized code in the Program RAM at startup. The data is transmitted via a conventional Serial Interface (IF) implementing a communication protocol such as Ethernet or USB. Table 1 shows sample specifications for a few selected available Nalu WFD ASICs, including AARDVARC and two other ultra-high timing precision, low power chips.

We have developed detailed physical modeling of light production and transport in DETECT2000[3] for multi-layer LYSO crystal arrays arranged in several geometries with one-to-one SiPM coupling at the bottom layer, as well as analytic and monte carlo modeling of sensor response and electronics for signal amplification, shaping and digitization using SPICE[4] and Matlab. Fig. 2 (top left) is the z vs x projection of a 8×8×3 (x,y,z) array of 2×2×7mm^3^ (2.2mm pitch) crystals showing the production of optical photons from a 511keV energy deposition originating at the center point of the top middle crystal; the top right plot is a “SiPMs’ eye” x-y view of the same data showing the lateral spread a shower has attained as it exits the crystal to be subsequently collected by a SiPM array. The outline of the upper crystal layers can be faintly seen from the exit face of the bottom layer. The bottom left plot shows the distribution of optical photon arrival times at the exit face of the crystal array produced by a 511keV shower. The characteristic LYSO rise time of slightly less than 2 ns is seen in the peaking bin in the bottom left plot; the ~43 ns LYSO 1/e decay constant can be seen in the bottom right plot showing the full decaying light curve of the same single event. We have developed a SPICE model of a Hamamatsu S13361 SiPM using as input the fully granular arrival time structure for individual optical photons such as shown in the bottom row of Fig. 2.

Finally, we have considered a baseline system-level architectural design based on a 64-channel WFD SymPET readout chip to understand the implications of systemic issues such as e.g. biasing, power and temperature control; channel and areal density, coincidence triggering network topology, combinatorics and rate including both signal and non-signal triggers from dark counts and other sources of noise; FPGA-based functionality including multi-channel and higher-level data analytics and fusion; data transmission rate requirements and reduction mechanisms at successively higher levels of systemic integration including considerations for export of final data (and metadata) for off-detector persistent storage and image reconstruction. We assume an idealized system geometry of a half spherical shell 230 mm in diameter (~9.1 in), instrumented with (256) 17.8×17.8 mm^2^ 8×8 SiPM arrays with 1-to-1 coupling to 2×2 mm^2^ crystals for a total of 16384 individual pixel channels; groups of readout chips are readout, triggered and controlled by “Level 1” FPGAs, with these L1 FPGAs in turn serviced by “Level 2” FPGAs which then feed into the “Top” system level, as shown in Fig. 3 (left). When a possible 511keV event is detected on a pixel, this information is sent to the L1 FPGA servicing the chip, which then passes it on to L2 and then to the Top level. The possible loci for a signal coincident with the original trigger is then calculated and that information sent back down to the L1 FPGA servicing the selected region of interest for triggering. Fig. 3 (left) diagrammatically shows this assumed topology at the L1 level and above, for (16) readout chips per L1, (16) L1 FPGAs, (2) L2 FPGAs and a single top layer. Using this topology along with a few variations, we have examined the relative rate of coincidence triggering relative to the ability to localize the possible L1 loci of a coincident partner to a triggering event as a function of the singles rate per crystal. Fig. 3 (right) shows the trigger rate relative to the assumption of “no triggers”, i.e. the linear “No trigger” line in the plot where no localization schema is adopted, for four varied L1-L2 topologies. Each of these L1-L2 localization schema shows an expected ~quadratic dependence, with the trigger rate decreasing as a function of increased L1 localization. However, for relatively very high numbers of L1s (such as 128), higher order effects than considered in our idealized analysis here will likely have at least some effect.

## Figures and Tables

**Figure 1.**
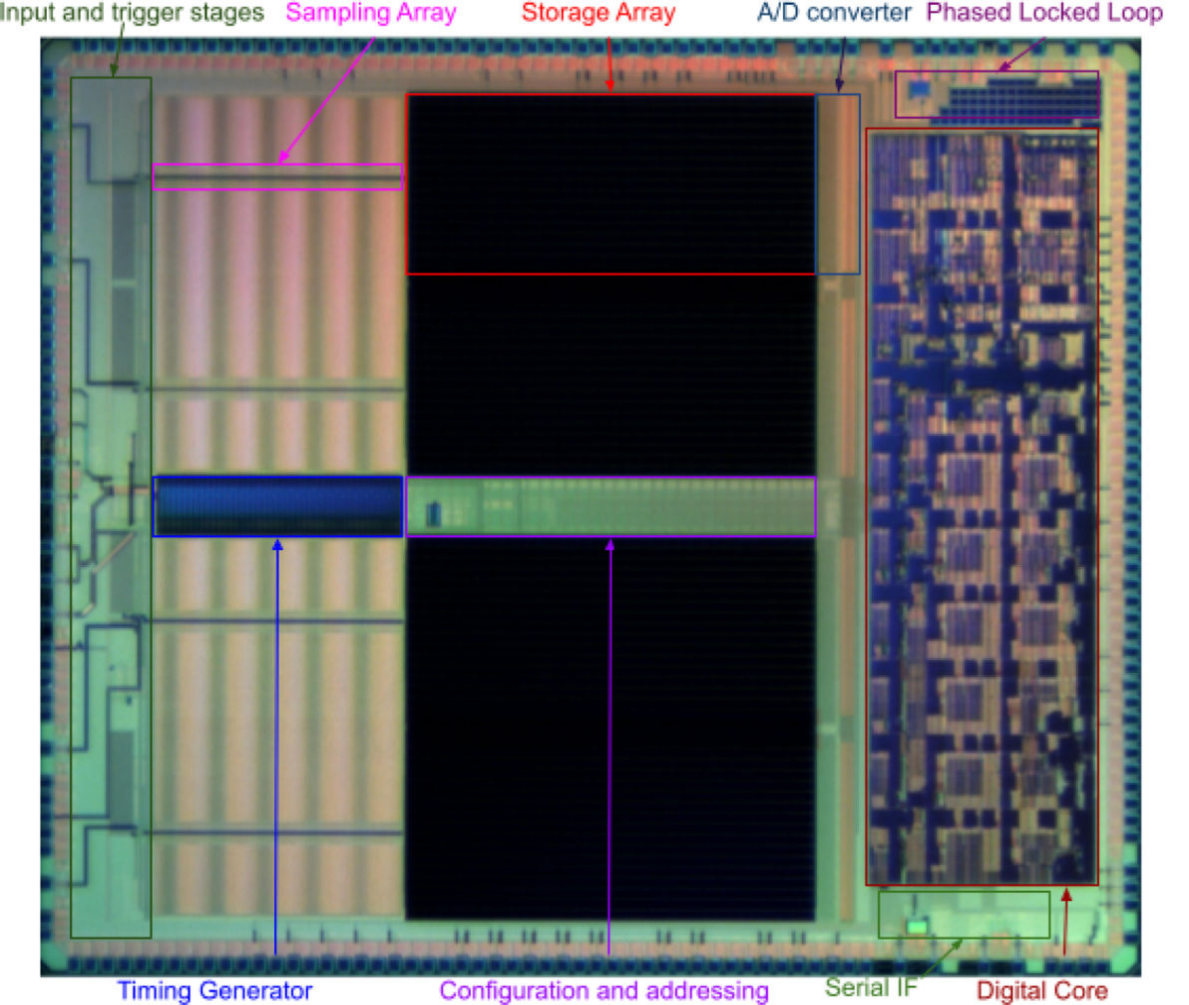
Micrograph of AARDVARC v3 in 130nm CMOS (5.88mm × 4.98mm, 29.3 mm^2^).

**Figure 2.**
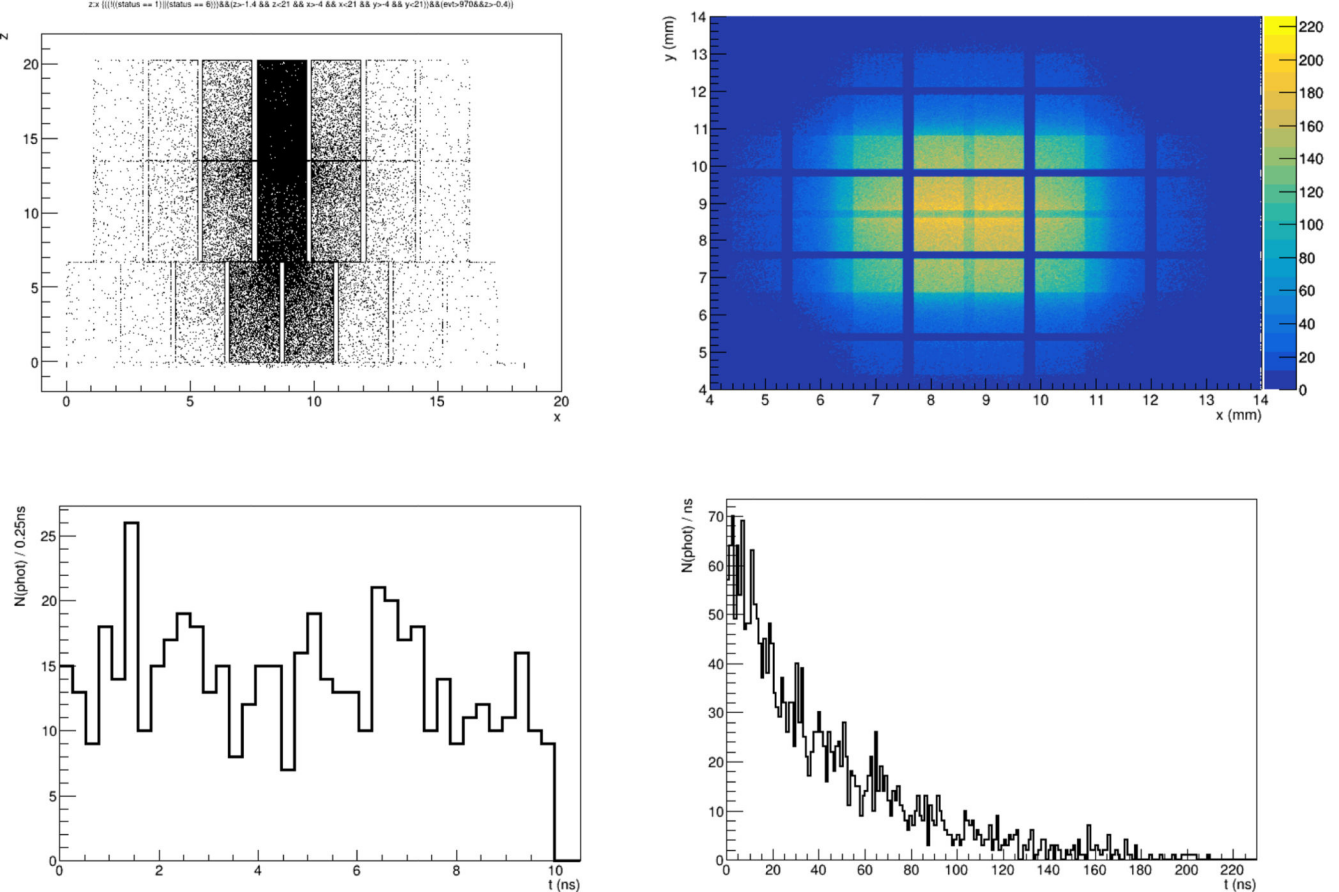
(top left) z vs x projection of a 8×8×3 (x,y,z) array of 2×2×7mm^3^ (2.2mm pitch) crystals showing the production of optical photons from a 511keV energy deposition originating at the center point of the top middle crystal; (top right) a “SiPMs’ eye” x-y view of the same data; (bottom left) optical photons arrival time at the SiPM-side exit of the array in the first 10ns; (bottom right) the full decaying LYSO light curve.

**Figure 3.**
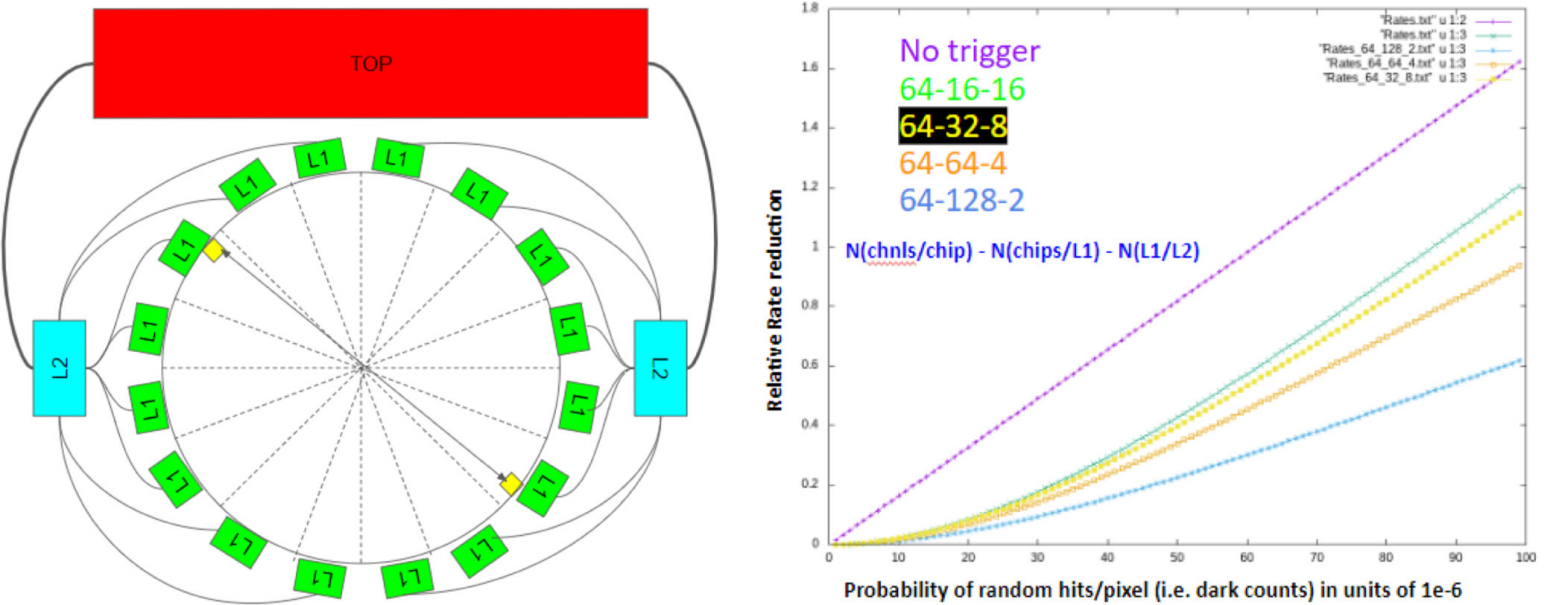
(left) xyz Block diagram of system-level triggering network topology; (right) Relative rate reduction for different L1-L2 topologies as a function of singles rate (dark counts) per pixel.

**Table 1.** Specifications for select available Nalu WFD ASICs.

Name	Tech. (nm)	# Ch	Speed (GSPS)	Buffer Depth (smpls)	Power/ch (mW)	Timing Jitter
AARDVARC	130/65	4/8	10–15	32768	70	10 ps, meas. pulse-to-pulse (<5 ps max poss.)
UDC	130	16	10	2000	15	5 ps
HPSoC	65	100+	10+	256	<5	<5 ps
STRAWZ	65	64	5	2048	<20	<10 ps
